# Effect of silver nanoparticles on gene transcription of land snail *Helix aspersa*

**DOI:** 10.1038/s41598-022-06090-1

**Published:** 2022-02-08

**Authors:** Faten Turki, Ridha Ben Younes, Mohsen Sakly, Khemais Ben Rhouma, José-Luis Martinez-Guitarte, Salem Amara

**Affiliations:** 1 Laboratory of Integrative Physiology, Faculty of Sciences of Bizerte, University of Carthage, 7021 Jarzouna, Tunisia; 2 Research Unit of Immuno-Microbiology Environmental and Carcinogenesis, Sciences Faculty of Bizerte, University of Carthage, Bizerte, Tunisia; 3 Grupo de Biología y Toxicología Ambiental, Departamento de Física Matemática y de Fluidos, Facultad de Ciencias, UNED, c/ Paseo de la Senda del Rey 9, 28040 Madrid, Spain; 4 Department of Natural and Applied Sciences in Afif, Faculty of Sciences and Humanities, Shaqra University, Afif, 11921 Saudi Arabia

**Keywords:** Environmental impact, Environmental sciences

## Abstract

Silver nanoparticles (Ag-NPs) are extremely useful in a diverse range of consumer goods. However, their impact on the environment is still under research, especially regarding the mechanisms involved in their effect. Aiming to provide some insight, the present work analyzes the transcriptional activity of six genes (*Hsp83*, *Hsp17.2*, *Hsp19.8*, *SOD Cu–Zn*, *Mn-SOD*, and *BPI*) in the terrestrial snail *Helix aspersa* in the presence of different concentrations of Ag-NPs. The animals were exposed for seven days to *Lactuca sativa* soaked for one hour in different concentrations of Ag-NPs (20, 50, 100 mg/L). The results revealed that the highest concentration tested of Ag-NPs (100 mg/L) led to a statistically significant induction of the *Hsp83* and *BPI* expression in the digestive gland compared to the control group. However, a trend to upregulation with no statistical significance was observed for all the genes in the digestive gland and the foot, while in the hemolymph, the trend was to downregulation. Ag-NPs affected the stress response and immunity under the tested conditions, although the impact was weak. It is necessary to explore longer exposure times to confirm that the effect can be maintained and impact on health. Our results highlight the usefulness of the terrestrial snail *Helix aspersa* as a bioindicator organism for silver nanoparticle pollution biomonitoring and, in particular, the use of molecular biomarkers of pollutant effect as candidates to be included in a multi-biomarker strategy.

## Introduction

Nanomaterials are extensively used in industry, and they present many shapes and sizes. One of them is the nanoparticles (NPs), with a size between 1 and 100 nm^1^. There are nanoparticles of different chemical nature, but one of the fastest-growing is silver nanoparticles (Ag-NPs) because of their thermoelectrical conductivity, catalytic activity, and nonlinear optical behavior. They are used in different industrial applications and biomedical facilities, such as imaging devices^2–5^. Additionally, since they have bactericidal properties, they are also present in many consumer goods^1,3,6–9^. The dark side of their extensive use is the insufficient knowledge about the impact that nanomaterials have on the environment. The last ten years have shown a linear increase in production and commercial use of nanomaterials, being used in many products such as personal care products, electronics, computers, or environmental remediation^5,10^. Subsequently, this extensive use has also caused an increasing presence in the soil as a component of fertilizers and biocides and other sources such as sewage sludge for agricultural or remediation purposes, industrial activities, or urban waste^11,12^, raising concerns about their impact on the living organisms.

Soil pollution is a central problem for human societies since it can affect the availability of soil for agriculture. It has been proposed that terrestrial gastropods can be good biomonitors and sentinels in soil pollution monitoring programs^13–19^. Among other, Pulmonata gastropods belonging to the *Helix* genus have been previously used to monitor trace metals, agrochemicals, urban pollution, and electromagnetic exposure^13,20–26^. The studied biological effects also include those affecting the growth, the reproductive capacity, and the induction of proteins involved in metal homeostasis and detoxification^26–28^. As happens in other organisms, the pollutants accumulated are metabolized and detoxified, being the digestive gland the primary target organ^22,25^. It has a crucial role in molluscan metabolism, showing a similar role observed in the vertebrate liver, such as the production of digestive enzymes, food storage, or excretion^29–31^. Furthermore, It has been described as a significant site for metal accumulation^14,32^, being a common target for biomarker measures to determine the damage caused by pollutants^14,26,29,33^. Finally, it has a relevant role in reactive oxygen species (ROS) generation^34^, a frequent response to the presence of pollutants. As a consequence, the analysis of the digestive gland is relevant in terms of the impact of metal pollution in physiological processes (e.g., growth, reproduction) and the accumulation of metals^20,26,35–37^.

There is a lack of information concerning the molecular response to toxicants in *Helix spp*., so any data will improve the battery of approaches to analyze toxicity. Oxidative and stress responses were the primary processes selected in this study since they are involved in the effects of several toxicants, including nanomaterials^19,38,39^. Oxidative stress implies ROS formation, which can damage DNA and lipids, among other effects. To prevent damage, several cell defense mechanisms manage and deactivate ROS^40^. Superoxide dismutases (SODs) are enzymes that can inactivate the superoxide radicals into molecular oxygen and hydrogen peroxide, i.e. Cu/Zn SOD and Mn-SOD^41^. The coding genes for both enzymes have been selected for analysis since it is expected that Ag-NPs could induce oxidative stress.

Heat shock proteins are the main proteins involved in the stress response, divided into several families depending on the molecular weight^42^. However, they are also involved in different cellular processes and diseases^43–45^. We have selected three genes coding for Hsp83, Hsp17.2, and Hsp19.8 to analyze the stress response in *H. aspersa* in the presence of Ag-NPs. The first one is a protein belonging to the Hsp90 family, related to cell survival, cell cycle control, hormone signaling, and apoptosis, among other physiological roles^46^. The two other genes selected code for small heat shock proteins. sHSPs participate in several biological processes, from the cell cycle to cell differentiation, from adaptation to stressful conditions and apoptosis^47^. These proteins show high homology in the alpha-crystallin domain (ACD) that characterizes them. In contrast to the ACD, the C-terminal and the N- terminal domains are less conserved among the various members and across the various species^48^. The relevance in toxicity of these proteins is that it has been observed that they are modulated by toxicants^49–51^, and are candidate molecular biomarkers.

Additionally, a sequence coding for an immune-related protein was selected. Bactericidal/permeability-increasing protein (BPI) is a protein involved in the response to bacteria, first described a long time ago and belonging to the lipid-binding serum glycoproteins^52^. It is part of the innate response and binds to lipopolysaccharides, killing Gram-negative bacteria, although it has also been associated with other immune roles such as chemoattraction or opsonization^53^. The gene was selected to study a putative mechanism of activation of the immunity that has been previously described^19^.

The toxicity of nanoparticles is a relatively recent topic that has received attention from researchers, mainly regarding the unknown effects they can have on the environment and health. Consequently, many studies have analyzed the impact of different types of nanoparticles in terms of size, composition, and shape, in a diversity of animals, especially from aquatic environments^54–56^. The situation is similar for Ag-NPs, which have been intensively studied because of their properties and their impact on ecosystems^56,57^, including soil ecosystems^12^. However, there are still gaps that need to be covered. Terrestrial mollusks are one of the animal groups which are poorly studied in response to nanoparticles. For example, it has been observed that *Theba pisana* shows oxidative stress, genotoxicity, and immunotoxicity in response to silver nanoparticles^19^. However, the molecular mechanisms involved are still poorly understood. Our study aims to provide additional information concerning the putative mechanisms and effects of Ag-NPs by evaluating in the terrestrial gastropod *Helix aspersa* by assessing the gene activity of six genes involved in the stress response and immunity. In addition, transcriptional activity was evaluated in different tissues to determine the extent of the damage and their sensitivity to the nanoparticles.

## Material and methods

### Chemicals

Ag-NPs < 100 nm (99.5% pure) were purchased from Sigma (Steinheim, Germany). They have been previously characterized^58^. Before use, each Ag-NP stock was mixed several times and an aliquot removed as a working solution that was sonicated 15 min in alternating cycles (2 × 30 s) ultrasonic sonicator. The physicochemical properties of Ag-NPs were confirmed by transmission electron microscopy (TEM) coupled with a microanalysis characterization (TECNAI G20, Ultra-Twin, FSB, Bizerte, Tunisia) and ultraviolet–visible (UV–Vis) spectroscopy (T60; PGInstruments, Leicestershire, UK). X-ray diffraction (XRD) characterization was performed using a D8 Advance diffractometer (Bruker, Bizerte), with analyses performed in Bragg–Brentano configuration at 40 kV and 40 mA.

### Animals

The snails were obtained from the Société d'élevage d'escargot—escargotière du nord (Bizerte, Tunisia), and they were initially collected from the mountains of Bizerte (Tunisia) to generate the farmed population.

Adult small gray specimens of *Helix aspersa* (mean weight 14.71 ± 0.2 g) were reared in a plastic box (55 × 39 × 25 cm) under controlled conditions regarding temperature (20 ± 2 °C), photoperiod (18/6 light/dark regime), and humidity (85%), according to De Vaufleury and Gimbert^59^. The lid of the box was perforated. Leaves of *Lactuca sativa* were administered ad libitum as food. One hundred and twenty one-year-old adult snails were randomly selected for the exposure experiment and starved for two days before starting the experiment.

### Experimental design

Foliar consumption of contaminated leaves is one of the primary natural pollutant exposure pathways in terrestrial snails. Therefore, a seven-day exposure to silver nanoparticles through contaminated *L. sativa* was performed. The animals were exposed three times over seven days to *L. sativa* previously soaked for 1 h in solutions of silver nanoparticles, following the method described by Dallinger et al.^60^**.** Three concentrations were utilized: 20, 50, and 100 mg/L to soak the lettuce. The lettuce leaves were covered by a solution of distilled water and nanoparticles at the concentrations used. After that, the leaves were shaked to remove the exccess of water and used to feed the animals.

The concentration of the nanoparticles in the lettuce leaves was measured by the Laboratory of the International Center for Environmental Technologies of Tunisia (CITET).Sample processing was performed with 50 g of leaf, which was dried at 40° C. The dried leaves were crushed in a pestle crusher in an inert atmosphere. A sample of 0.5 g was treated at 450 °C for 3 h and cool down to room temperature. The residue was collected with demineralized water and 5 ml of hydrofluoric acid and 1.5 ml of perchloric acid were added. The sample was heated at 160 °C until the solution was completely evaporated. Then, 1 ml of hydrochloric acid and deionized water were added to disolve the residue.The silver assay to detect the nanoparticles was performed by emission spectroscopy optics with plasma induced by high frequency (ICP-OES) according to standard NF EN ISO 11,885. The obtained results are showed in Table S1. Four boxes for each repeat were utilized (control, exposed 1, exposed 2, exposed 3), and ten animals were added to each box (plastic box dimensions, 55 × 39 × 25 cm). All the groups were held at a controlled temperature, photoperiod, and humidity (see above). After seven days, all animals per box were sampled. Each specimen underwent hemolymph sampling, performed by a sterilized hypodermic syringe (3 ml) through a small hole created on the shell at the hemocoel level, according to Regoli et al.^15^.

Samples were pooled, and hemolymph was immediately frozen to be later processed. Then, each snail underwent cold anesthesia (4 °C for 20 min) and was culled. Next, the digestive gland and the foot were dissected, frozen using dry ice, homogenized in 300 μL TRIzol, and stored at – 80 °C until RNA was isolated.

### Identification of genes

A transcriptome project from the SRA database was downloaded (SRX1015093, *Helix aspersa* tentacle transcriptome) and assembled by using OMICS Box software (OmicsBox—Bioinformatics made easy (Version 1.4.11). BioBam Bioinformatics. March 3, 2019. https://www.biobam.com/omicsbox). Afterward, the assembled sequences were compared to the GenBank database with the BLAST software's functional analysis tool. Seven genes were selected to perform the study. Three of them were related to stress response (*Hsp 17.2*, *Hsp19.8*, *Hsp83*), two with oxidative stress (*SOD CuZn*, *Mn-SOD*), one with the immune system (*BPI*), and the last one was chosen as a reference gene (*GAPDH*). The sequences and the open reading frame (ORF) coding for the proteins are shown in the supplementary material.

### RNA isolation and complementary DNA (cDNA) synthesis

Each homogenate sample in TRIzol was used to isolate total RNA following the manufacturer's instructions. Briefly, 0.2 volumes of chloroform were added to the 300 μL homogenate and thoroughly mixed, incubated for 2–3 min at room temperature, and centrifuged at 10,000 rpm at 4 °C. The aqueous upper phase was recovered, and RNA was precipitated with isopropanol and washed with 70% ethanol. DNAse treatment was performed using RNase-free DNase (Roche, Germany), followed by a phenol/chloroform/isoamyl extraction with Phase Lock Light tubes (5prime, Spain). The isolated RNA was resuspended in 100 μL diethylpyrocarbonate (DEPC)-treated water, quantified by UV spectrometry (Biphotometer, Eppendorf), and stored at − 80 °C.

Synthesis of cDNA was performed with 8 μg RNA in a final volume of 40 μL. MMLV (Invitrogen, Germany) was used as an enzyme, and the reaction was carried out following the manufacturer's instructions. The cDNA was stored at -20 °C until use in RT-PCR^61^.

### Real time-PCR

The cDNA (0.6 μL/well) was used as a template for RT-PCR. Primers were designed by using the Primer-BLAST tool^62^ with the isolated sequences in this article. Primers were obtained from Macrogen (Korea). The RT-PCR was performed with a CFX96 thermocycler (Bio-Rad, USA) by using 0.5 unit DNA polymerase (Biotools, Spain), 0.4 mM dNTPs, and 0.5X EvaGreen (Biotium, USA). The thermal cycling program included an initial denaturation at 95 °C for 30 s followed by 40 cycles of 95 °C denaturation for 15 s, 50 °C annealing for 15 s, and 72 °C elongation for 30 s. The presence of a single peak was confirmed by doing a melting curve. The reference gene used was *glyceraldehyde-3-phosphate dehydrogenase* (*GAPDH*). The genes' efficiency was obtained by doing five different 1:2 dilutions by duplicate to get the curve. Each sample was run in duplicate wells, and two independent replicates were used for each experiment. Primer sequences and efficiencies are listed in Table 1. Bio-Rad CFX Maestro software was used to analyze and determine total mRNA levels of normalized gene expression (2^−ΔΔCq^)^61^.

**Table 1:** Primer sequences and PCR efficiency for the genes used in this study.

Primer	Primer sequence	Efficiency (%)
Hsp83 F	5′-TGG GAA GAC CAC TTG GCA GT-3′	82.9
Hsp83 R	5′-CAA ATG GCG CCC TCT TTG GG-3′	
Hsp17.2 F	5′-TGA CCC TAA GTC GGT GAC CT-3′	79.2
Hsp17.2 R	5′-AAC TCA ATG GGG ATT GCC GT-3′	
Hsp19.8 F	5′-ACG GCT GTC TCT GAG GTT TG-3′	85.3
Hsp19.8 R	5′-GTG TGT TTG GCA TGC ACC AT-3′	
Cu–Zn SOD F	5′-TAG TTG CCA ATG CTG ACG GT-3′	93.3
Cu–Zn SOD R	5′-GGA CCA CAA CAC TAC GAC CA-3′	
Mn-SOD F	5′-ACC CTC TTC AAG CGA CAA CA-3′	92.5
Mn-SOD R	5′-CTC CGA CAC GTC TTT CCA GT-3′	
BPI F	5′-AGC TTG ACA ATG ACA GGG CA-3′	76.9
BPI R	5′-GGT CAA CAC GGT CTC CAC AA-3′	
GAPDH F	5′-CGT CCG CAG CTC AAT CTT TG-3′	86.5
GAPDH R	5′-CAA CCA CCC GGT TGG AGT AA-3′	

### Statistical analysis

Statistical analysis was done using XLSTAT 2020 (Addinsoft, France). The Shapiro–Wilk test was performed to test whether the data were normally distributed and to obtain the variance homogeneity. For normally distributed data, significant differences were determined using analysis of variance (ANOVA). The non-normally distributed data were analyzed with the nonparametric Kruskal–Wallis test. Statistical significance was set at *p* ≤ 0.05. Graphs were prepared using using Excel (Microsoft, USA).

### Ethical approval

Invertebrates used in the present work did not require Ethical Aproval from the Ethics Commite. The animals were grown following the higher standards and taking care of their welfare.

## Results

### Sequence characterization

**Figure 1:**
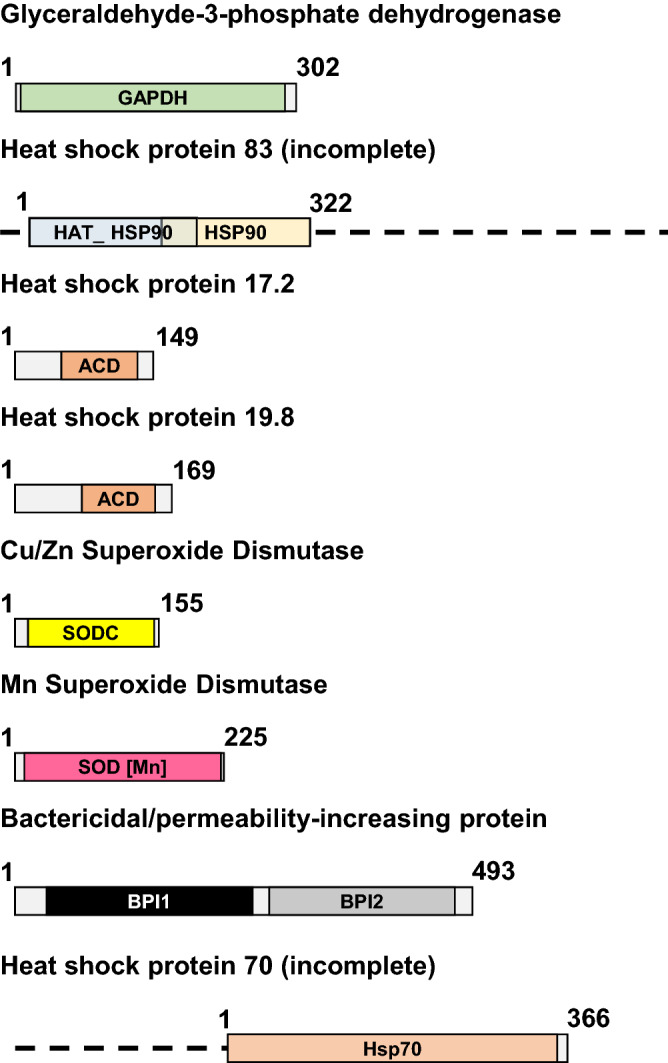
Scheme of the ORFs corresponding to the sequences identified. The numbers indicate the amino acid size of the ORF. For each ORF, the characteristic domains of each protein are indicated to indicate homology. The motifs are shown in the region of the protein where they are located. GAPDH: glyceraldehyde-3-phosphate dehydrogenase domain; HATPase_Hsp90: histidine kinase-like ATPase domain; Hsp90: heat shock protein 90 protein domain, ACD: alpha-crystallin domain; SODC: copper/zinc superoxide dismutase domain; SOD [Mn]: manganese superoxide dismutase domain; BPI1: BPI/LBP/CETP N-terminal domain; BPI2: BPI/LBP/CETP C-terminal domain. The figure was drawn with Powerpoint (Microsoft, USA).

Seven sequences were identified by analyzing a transcriptome project from the database (SRX1015093, *Helix aspersa* tentacle transcriptome). The sequences size and the ORF size are shown in Table 2. Comparing each ORF to the GenBank database showed that six of the sequences had a complete ORF while the seventh, corresponding to Hsp83, was only a partial sequence of the protein. The BLAST comparison showed that one of the sequences corresponds to the glyceraldehyde-3-phosphate dehydrogenase since it has homology to that protein in the gastropod *Cepaea nemoralis*. Furthermore, the protein coded showed the characteristic GAPDH domain of these enzymes (Fig. 1). This gene is commonly used as a reference gene since it is considered housekeeping by its implication in glycolysis.

**Table 2:** Sequences characteristics and identification.

Gene	DNA size (bp)	ORF (aa)	Homology	Identity/similarity
GAPDH	1267	302	glyceraldehyde-3-phosphate dehydrogenase [*Cepaea nemoralis*] AXI69348	99%/100%
Hsp83 (partial)	966	322	heat shock protein 83 [*Aplysia californica*] XP_005102885	82%/92%
Hsp17.2	1690	149	heat shock protein 17 [*Physella acuta*] AXN72715	68%/79%
Hsp19.8	1014	169	heat shock protein 20.4 [*Physella acuta*] AXN72713	77%/90%
Cu/Zn-SOD	1351	155	superoxide dismutase [*Aplysia californica*] NP_001191510	77%/87%
Mn-SOD	944	225	superoxide dismutase [Mn], mitochondrial [*Aplysia californica*] XP_005104703	83%/88%
BPI	2138	493	bactericidal permeability-increasing protein [*Aplysia californica*] XP_005112375	46%/68%

The other six genes also showed high homology with proteins from other gastropods, four of them with *Aplysia californica* proteins (*Hsp83*, *Cu/Zn-SOD*, *Mn-SOD*, *BPI*) and the two other with *Physella acuta* (*Hsp17.2* and *Hsp19.8*). The presence of the characteristic domains for each protein (Fig. 1) confirmed the identity of each gene. The name of the small heat shock proteins was assigned following the consensus of using the molecular weight. The homology with other small HSPs is mainly in the alpha-crystallin domain, challenging to know the exact equivalence with the sHSPs from other species.

### Gene expression analysis

It has been suggested that the silver nanoparticles affect oxidative stress and stress response, so five genes involved in those processes were analyzed to elucidate the putative impact and mechanisms involved in *H. aspersa*. Furthermore, to evaluate the impact in different tissues, the mRNA levels were analyzed in the hemolymph, digestive gland, and foot.

#### Hemolymph

**Figure 2:**
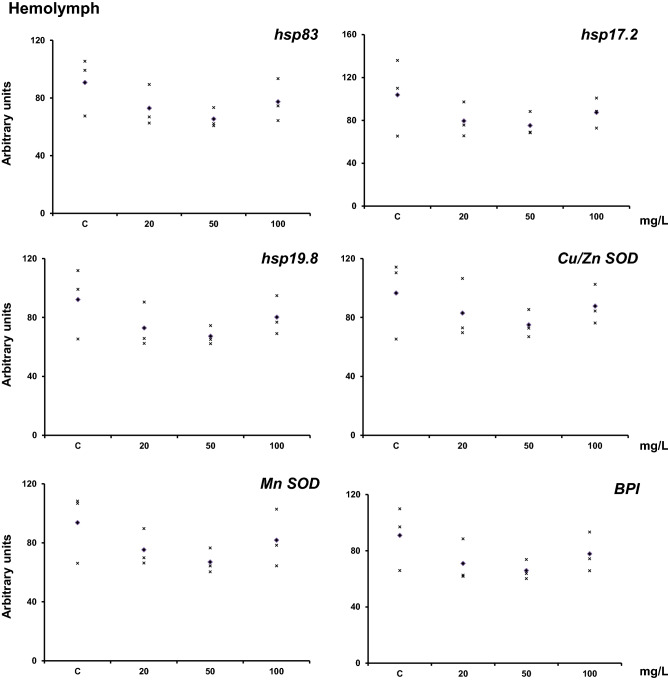
Transcriptional activity of *hsp83*, *hsp17.2*, *hsp19.8*, *Cu–Zn SOD*, *Mn-SOD*, and *BPI* in adult *H. aspersa* hemolymph samples after in vivo exposure to Ag-NPs at (20 ± 2 °C) for 7 days. Animals were fed with lettuce soaked for one hour with the concentrations of silver nanoparticles indicated on the x-axis. Levels of mRNA were normalized using GAPDH as the reference gene. Three experiments were carried out, and the data are shown for each experiment (x). The diamond indicates the mean. The figure was drawn with Excel (Microsoft, USA).

The mRNA levels in hemolymph, reflecting the response of its cellular component, for the six genes analyzed had a trend to downregulation with lower mean levels for the two first concentrations of Ag-NPs (Fig. 2). However, the distribution of the data did not provide a statistically significant difference.

#### Digestive gland

**Figure 3:**
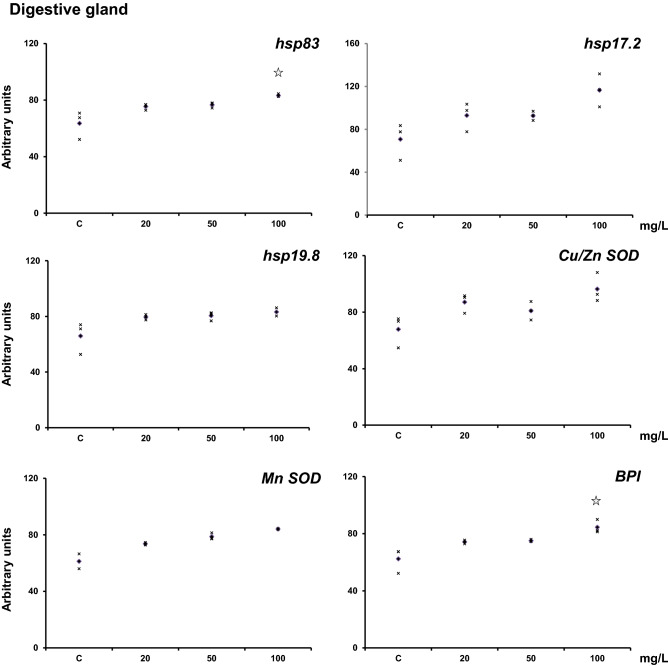
Transcriptional activity of *hsp83*, *hsp17.2*, *hsp19.8*, *Cu–Zn SOD*, *Mn-SOD*, and *BPI* in the digestive gland of adult H. aspersa. Animals were exposed to Ag-NPs at (20 ± 2 °C) for 7 days by feeding them with lettuce soaked in silver nanoparticles. Levels of mRNA were normalized using GAPDH as a reference gene. Three experiments were carried out, and the data are shown for each experiment (x). The diamond indicates the mean. The star indicates statistically significant differences against control animals (*p* < 0.05). The figure was drawn with Excel (Microsoft, USA).

Contrary to that observed in hemolymph, the digestive gland showed a trend towards increasing the six genes' mRNA levels analyzed after exposure to Ag-NPs (Fig. 3). The trend suggests a dose–response effect with a higher increase with a higher concentration of Ag-NPs. Similarly to that observed for hemolymph, the statistics did not show any difference except for the highest concentration of *Hsp83* with control.

#### Foot

**Figure 4:**
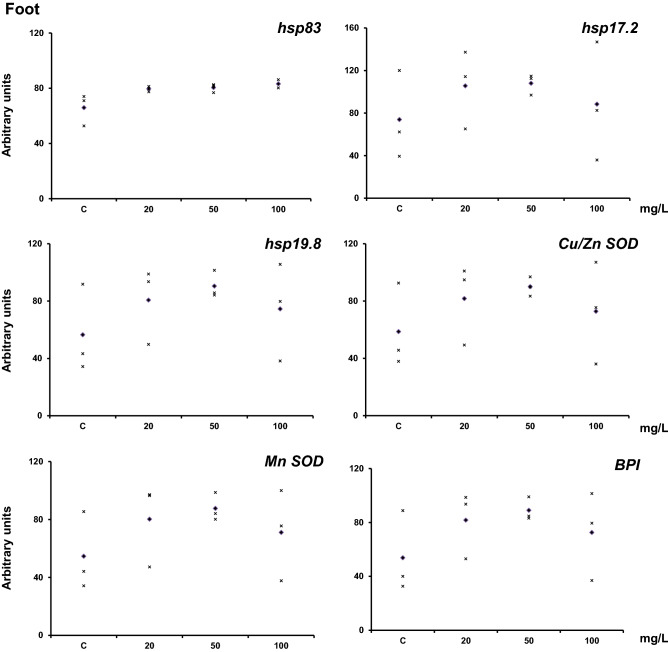
mRNA levels of *hsp83*, *hsp17.2*, *hsp19.8*, *Cu–Zn SOD*, *Mn-SOD*, and *BPI* in adult *H. aspersa* foot. The animals were exposed to Ag-NPs at (20 ± 2 °C) for 7 days by feeding them with lettuce soaked for one hour in the indicated silver nanoparticle concentrations. The analysis was performed by Real-Time PCR using GAPDH as a reference gene. Three experiments were carried out, and the data are shown for each experiment (x). The diamond indicates the mean. Figures were drawn with Excel (Microsoft, USA).

The response of the genes in the foot showed a different pattern to that observed in the hemolymph and digestive gland (Fig. 4). It was opposite to that observed in hemolymph with a trend to an inverted U-shape depending on the concentration. However, the statistics did not show any differences between the samples because of the data distribution.

## Discussion

In this work, we analyzed the putative alteration of transcriptional activity by silver nanoparticles focusing on the transcriptional activity of genes related to the stress response, the oxidative stress, and the immunity of *Helix aspersa*. This snail is a bioindicator of toxicity species, but it is poorly understood at the molecular level. We identified seven sequences that code for glyceraldehyde-3-phosphate dehydrogenase (GAPDH), three heat shock proteins (Hsp83, Hsp17.2, Hsp19.8), two superoxide dismutase (SOD Cu/Zn, Mn-SOD), and one immune protein (BIP). They showed similarity to those found in other gastropods, mainly with aquatic species, since they are the most studied at the molecular level. Furthermore, all the sequences presented the characteristic domains to be considered homologous in *H. aspersa*. With little previous information, any addition to the set of genes of these species would favor future research into the molecular mechanisms of toxicants. On the other hand, these genes allowed us to analyze the putative molecular mechanisms working in the cell to respond to Ag-NPs.

Silver nanoparticles have been extensively studied in different organisms, but there are still gaps regarding the mechanisms and the effect that they can have in invertebrates and, precisely, in terrestrial gastropods. It is known that they can induce oxidative damage, which activates the antioxidant defense systems, including enzymes and low-molecular non-enzymatic antioxidants^63,64^. It has been observed in *C. elegans* that Ag-NPs activate the transcriptional activity of *sod-1* (also known as *Cu–Zn SOD*)^65^, while upregulation of *Sod2* (also known as *Mn-SOD*) has been observed in *Drosophila melanogaster*, also affecting hemocytes^66^. The induction of both SOD genes has also been described in the aquatic larvae of *Chironomus riparius* in response to Ag-NPs^67^. In a species closer to *H. aspersa*, the freshwater snail *Bellamya aeruginosa*, Ag-NPs accumulated, from highest accumulation to lowest, in the hepatopancreas, gonad, digestive tract, and foot, causing oxidative stress^68^. Although our results did not show upregulation for either superoxide dismutase gene, suggesting that there is no activation of the oxidative stress response in the long term, there is also a previous report in the copepod *Calanus finmarchicus* where no induction of the *SOD* gene was observed^69^. Several reasons could explain the different responses observed. One factor could be the nanoparticles used, which can vary in size, form, composition, and concentration. Another factor to consider is the experimental approach. For *D. melanogaster* and *C. elegans*, the nanoparticles were in the food and the culture medium, respectively, but it is important to consider the form of culture. *D. melanogaster* larvae are usually in frequent contact with the food while *H. aspersa* received the food three times per week. Therefore, comparing regular exposure to an intermittent exposure approach could explain the lower induction of the response, since it could reflect a lower intake in the snail than *D. melanogaster* and *C. elegans*. Another factor to consider is that the amount of silver nanoparticles in the soaked lettuce leaves depends on the quantity adsorbed by the leave. Thus, the snails' actual dose could be too low to induce the robust oxidative stress response observed in other animals. However, it is worth remembering that the exposure procedure used in this work is closer to the situation that could be found in the wild, by using water polluted with nanoparticles or recycled water for agriculture.

Focusing attention on the stress response, we observed an upregulation in the digestive gland for the highest concentration used for *hsp83* but not the response of the other two genes tested. In *C. finmarchius*, upregulation of *hsp90* was observed after exposure to silver nanoparticles^69^, which agrees with our observation, but no impact was observed in *Gammarus fossarum* for the same gene^70^. It is relevant to mention that *HSP90AA1* has been reported as the top upregulated gene in the heat shock protein family in human epithelial cells^71^. The increase in Hps83 cannot be ignored since the Hsp90 proteins are involved in other processes such as hormonal signaling or apoptosis^46^. A deeper analysis, including different times and additional genes, could show additional effects of Ag-NPs in other cell processes, which justify the higher mRNA levels. In addition, the increase was only observed in the digestive gland. Its role as a detoxification organ in gastropods could explain this finding. The absence of response in the foot and hemolymph samples could be due to low levels, since the hemolymph could be acting as a transporter to the digestive gland, while the foot could not absorb them when in contact with the leaves. Quantification of the nanoparticles in both tissues would help explain the reason for a different response concerning *hsp83*.

Concerning the small heat shock proteins, Roh et al. described that no significant changes were observed in transcriptional activity of three genes coding small heat shock proteins, *hsp16.1*, *hsp16.2*, and *hsp16.41*, analyzed in *C. elegans*^72^ in response to Ag-NPs. This agrees with our observations, but it is hard to find any equivalence between the genes. sHSPs are highly conserved in their specific domain, the alpha-crystallin domain. However, they show high variability in the C-terminal and N-terminal domains, so it is difficult to know the equivalence between proteins from different species. Furthermore, the number of sHSPs in each species is highly variable^73,74^, increasing the complexity of the analysis. Nevertheless, the data obtained are in line with previous results, so it is likely that sHSPs were not affected. However, an earlier activation time point cannot be discarded since variation between the response activity depends on the small heat shock protein^44^. Furthermore, the lack of induction could suggest that Ag-NPs do not alter the structure of proteins, since the role of sHSPs is to maintain protein homeostasis by binding proteins in non-native conformations, thereby preventing substrate aggregation^75^.

Taking together the results on stress responses in invertebrates, they seem to be variable. In other species, such as *D. melanogaster*, it has been reported that silver nanoparticles can induce HSP70^76^ and, similarly, *hsp70* is also upregulated in the dipteran *Chironomus riparius*^77^. The effects may depend on the type of nanoparticle, the duration of exposure, the method of exposure, and the physiology of the organism. Therefore, it would be necessary to use a multispecies approach with the same nanoparticles to perform a comparative analysis with less variability between experimental approaches. With the present knowledge, it is not easy to ensure that the Ag-NPs used in this study produce a similar response to that observed in other organisms. More extended experiments would also help to define the impact in the long term.

The analysis of the transcriptional activity of the *BPI* gene allowed us to check the immune response to bacteria. To our knowledge, there is no previous report for this protein in invertebrates concerning the toxicity of silver nanoparticles. Our observation shows that it was increased in the digestive gland at the highest concentration used, with no changes in the rest of the tissues and conditions tested. The impact of Ag-NPs on immunity has been described for sea urchin^78^, and they can evoke immune responses in terrestrial and freshwater annelids^79^. However, in that case, the authors focused on the role of the cells involved in the immune response. BPI alterations have been described for a nematode-based molluscicide in the kidney and the gills of *Pomacea canaliculata*^80^. The increase was related to the presence of the nematode in the organs, as other organs with no nematodes, such as the mantle, did not show increased BPI transcriptional activity. On the other hand, it has been established that BPI is in a family of proteins in mollusks with more than one representative, and they are expressed in a tissue-specific way^81^. In addition, BPI has been described as a multifunctional protein since it can work also as an anti-angiogenic, chemoattractant, or opsonization agent^53^. Therefore, the observed change in BPI transcriptional activity in *H. aspersa* could reflect a response not related directly to its anti-microbial activity. However, the activation of transcription in the digestive gland could reflect a response to the presence of Ag-NPs, which were identified as strange to the organisms, mimicking bacteria. On the other hand, at the physiological level, the modulation of BPI activity by silver nanoparticles could affect the typical response to microbes by altering BPI expression and, by extension, to other parasites such as nematodes since, in some cases, they use bacteria as secondary elements to kill snails. In this sense, it could activate the response and act by preventing the activity of bacteria in the snail. Additional research would be needed to elucidate the putative impact of silver nanoparticles on the immune system and determine whether it is beneficial or harmful in the long term for the population.

Finally, the different tissues used only showed a statistically significant result for the highest concentration of Ag-NPs in the digestive gland. Nonetheless, some trends suggested differential sensitivity to Ag-NPs for each tissue with a different pattern of response depending on the dose. In comparison, hemolymph samples showed an inverted U-shape while the foot showed a U-shape response. The digestive gland showed a more typical response with increasing transcriptional activity at higher concentrations. It has been previously described that Ag-NPs can reach different tissues to varying degrees; the hepatopancreas (also known as the digestive gland) is the organ that receives the highest concentrations^68^. Therefore, it would be a factor to consider in the response of each organ to the presence of Ag-NPs. In our case, the results seem to confirm that the digestive gland is the most affected organ, as expected, since it is the primary site of biotransformation of xenobiotic and oxy-radical-generating enzymes in snails^82^. However, more studies are required to determine the extent of the response and explain why the foot and the hemolymph samples showed inverse responses at moderate concentrations. This is critical because hemocytes usually transport pollutants to the digestive gland^22,25^, so it would be expected to see some response in the hemolymph samples. However, the exposure method used could have some influence. The exposure consisted of three intermittent exposures, so the increase in Ag-NP levels in hemolymph samples could be temporary, with time to recover normal conditions by the time of analysis. Additional experiments with different time points before and immediately after feeding could help understand the dynamics of Ag-NP and tissue responses.

## Conclusion

The analysis at the molecular level of the impact of silver nanoparticles in the terrestrial gastropod *Helix aspersa* shows that the digestive gland was affected by the highest concentration tested. Oxidative stress induction was not observed, while the stress response showed a weak activation of *hsp90* at the highest concentration tested in the digestive gland. The alteration in *BPI* transcriptional activity could have a critical impact since it is involved in responding to bacterial and parasite responses, affecting the population's health. Overall, the results suggest a weak response to Ag-NPs. However, the method of exposure and the analysis could be factors to consider the effect in the tissues and organs since different trends could be observed. Further research involving different time-point analyses would help to elucidate the response and the relevance of mimicking wild conditions by using food contaminated with Ag-NPs as an approach. The description of six new genes in *H. aspersa* extends the number of putative biomarkers to analyze the impact of toxicants in this species and provides new tools for assessing toxicity by combining them with traditional ecology-relevant endpoints.

## Supplementary Information

Supplementary Information.

## Data Availability

All sequence data used in the present article are from public database and they have been included in the supplementary materials.
