# Supplementary material for: Effect of silver nanoparticles on gene transcription of land snail Helix aspersa
Source: Sci Rep. 2022 Feb 8;12:2078. doi: 10.1038/s41598-022-06090-1 (PMC8826417; doi:10.1038/s41598-022-06090-1)
Supplement: Supplementary file 1 — Supplementary Information. [file 41598_2022_6090_MOESM1_ESM.docx]

**Supplementary material**

**Effect of silver nanoparticles on gene transcription of land snail *Helix aspersa***

**Faten Turki^1^, Ridha Ben Younes^2^, Mohsen Sakly^1^ , Khemais Ben Rhouma¹, José-Luis Martinez-Guitarte^3^ and Salem Amara^1,4^.**

**^1^ University of Carthage, Faculty of Sciences of Bizerte, Laboratory of Integrative Physiology, Jarzouna 7021, Tunisia.**

**^2^Research Unit of Immuno-Microbiology Environmental and Carcinogensis, Sciences Faculty of Bizerte, University of Carthage, Bizerte, Tunisia.**

**^3^Grupo de Biología y Toxicología Ambiental. Departamento de Física Matemática y de Fluidos. Facultad de Ciencias – UNED. c/ Paseo de la Senda del Rey 9, Madrid 28040**

**^4^Shaqra University, Faculty of Sciences and Humanities, Department of Natural and Applied Sciences in Afif, Afif 11921, Saudi Arabia.**

**Table S1**

| **DETERMINATION OF TRACED METALS** | | | | | | | | |
| --- | --- | --- | --- | --- | --- | --- | --- | --- |
| **Determination of 33 elements by atomic emission spectroscopy with induction coupled plasma.** | | | | | | | | |
| **EN ISO 11885-August 2007** | | | | | | | | |
| **Samples** | **Elements** | **P.E (g)** | **Vm (ml)** | **Dil f** | **%MS** | **Ci (mg/L)** | **Bl (mg/L)** | **Cf (mg/Kg MS)** |
| **0 – 1** | Ag | 1,17 | 50 | 1 | 51 | 0,000 | 0 | 0 |
| **0 – 2** | Ag | 0,9508 | 50 | 1 | 63 | 0,000 | 0 | 0 |
| **20 – 1** | Ag | 1,1065 | 50 | 1 | 53 | 0,149 | 0 | 12,7036 |
| **20 – 2** | Ag | 1,1682 | 50 | 1 | 88 | 0,268 | 0 | 13,0348 |
| **50 – 1** | Ag | 1,1909 | 50 | 1 | 49 | 0,218 | 0 | 18,6790 |
| **50 – 2** | Ag | 1,173 | 50 | 1 | 52 | 0,198 | 0 | 16,2305 |
| **100 – 1** | Ag | 1,664 | 50 | 1 | 50 | 0,311 | 0 | 18,6899 |
| **100 – 2** | Ag | 1,1245 | 50 | 1 | 54 | 0,289 | 0 | 23,7965 |
|  |  |  |  |  |  |  |  |  |
| **The formula:** | | | | | | | | |
| **Cf (mg/kg)= (Ci-blanc)x f x Vm /(PE*MS/100)** | | | | | | | | |
| **C_f_**: The final content of the metal in mg/L. | | | | | | | | |
| **Ci**: The content obtained by the device. | | | | | | | | |
| **f**: possible dilution factor. | | | | | | | | |
| **PE**: Volume of the test sample | | | | | | | | |
| **Vm**: The volume in ml of the sample to be assayed | | | | | | | | |
| **MS**: dry matter | | | | | | | | |

**Figure S1: DNA and protein sequences of the genes identified. ORFs are indicated with bold and underlined text.**

**Glyceraldehyde-3-phosphate dehydrogenase GAPDH (1267 bp)**

TTCCGTCATCATTCTCTTAAGCCGGGTCGCTGAGATACCTAAGCAAAAACAAA**ATGCCTGAACTCAAAGTTGGAATCAATGGATTTGGACGTATTGGTCGTTTGACCTTGCGCGCTGCCCTTGAGAAGAAGGTCAATGTTGTAGCCGTAAACGACCCATTCATCAGCCTGGAGTACATGGTGTACATGTTCAAGTACGACTCCACCCACGGCCGTTACAAGGGCACAGTAAAAGAGAACAGCGGAAAGTTGGAGATTGATGGCCACCTCATCACTGTGTTTGCCGAGAAAGACCCTGCTGCTATTGACTGGAAATCAACAGGTGCTAACTACGTCGTTGAATCAACAGGAGTGTTCACAACAGCAGATAAGGCTAATGTACATATCAAGAGTGGAGGAGCCAGTAAGGTGGTCATCTCCGCACCTTCCGCCGACGCCCCCATGTTTGTGCTGGGCGTCAATGAAGATAAGTACACCAAGGATCTGACCGTGGTCAGCAACGCTTCCTGCACCACCAACTGCCTGGCCCCCCTGGTCAAAGTCATCAATGACAAGTTTGGAATTGTTGAGGGGCTGATGACGACCGTGCATGCCACAACCGCCACTCAGAAGACCGTCGATGGCCCCAGCAACAAGGACTGGCGTGGAGGTCGTGGAGCTGCCCAGAACATCATCCCATCTTCGACAGGTGCCGCTAAAGCTGTGGGAAAAGTCATCCCTGCCGTTAACAACAAACTGACTGGAATGGCTTTCCGTGTACCAGTTGCTGACGTGTCAGTCGTGGACCTCACTGTTCGACTGGAGAAAGGGGCATCCTATGAGGAGATCAAGAAGGCAGTCAAAGATGCCTCTGAGACATCCTTGAAGGGTATCCTGGGCTACACCGAAGATGATGTTGTGTCCTCGGACTTCCTGGCGACGTCCGCAGCTCAATCTTTGATGCCAATGCTGGCATTGCTT**TGAACAGCAACTTTGTGAAACTCGTTGCATGGTATGACAATGAATATGGTTACTCCAACCGGGTGGTTGAGCTCATCCAGCACATGTACAAAGTGGACAACCAGTAAATGAAGCTTCACATGGTTATCTACACCAGCGCCTGACGCTGAACAACTTGAAGACTATCATCCTTATGTCCCACCGAGAAACTTGCAACTTGCAGAGATTGTCATGTCACCAGAGATCTCGATTAAGTATTAGTTTTGATAAGTCATGTTGAGCCAGATAAATGTGATCACTGGTAATAAATGTATAGAACCATGAAAAAA

**Protein (302 aa)**

MPELKVGINGFGRIGRLTLRAALEKKVNVVAVNDPFISLEYMVYMFKYDSTHGRYKGTVKENSGKLEIDGHLITVFAEKDPAAIDWKSTGANYVVESTGVFTTADKANVHIKSGGASKVVISAPSADAPMFVLGVNEDKYTKDLTVVSNASCTTNCLAPLVKVINDKFGIVEGLMTTVHATTATQKTVDGPSNKDWRGGRGAAQNIIPSSTGAAKAVGKVIPAVNNKLTGMAFRVPVADVSVVDLTVRLEKGASYEEIKKAVKDASETSLKGILGYTEDDVVSSDFLATSAAQSLMPMLALL

**Heat shock protein 83 (966 bp, incomplete)**

**AACACCTTCTACAGCAACAAGGAAATTTTCCTTCGTGAGTTGATTTCCAATGCATCTGATGCTCTTGACAAAATTCGCTACGAGTCTCTCACAGATCCAAGTAAACTGGACACGGGGAAAGAGCTACACATCAGAATCATTCCAGACAAGGAGAACAAGACCCTCACTATTGAAGACACTGGAATTGGAATGACCAAGGCTGACCTTGTCAATAACCTGGGCACTATTGCCAAATCTGGCACCAAAGCCTTCATGGAAGCCTTGCAGGCAGGAGCTGACATCTCCATGATCGGACAGTTTGGTGTAGGTTTCTATTCAGCATACCTTGTGGCAGATCGTGTGGTTGTGGATTCCAAAAATAATGATGATGAGCAGTACACCTGGCAGTCTTCAGCCGGTGGGACATTTACTGTAGAGCCTTCAACCAGTGCTCCTCTTTCACGTGGTACCAGGGTCACTTTGTATCTCAAAGAAGATCAGCTGGAGTACTTGGAAGAGAGGAAGATCAAGGATGTAATCAAGAAACACAGCCAGTTTATTGGCTACCCAATCAAACTACTGGTTGAGAAGGAGCGCGACAAAGAAGTGTCTGATGATGAAGAGGAAGAGAAGAAAGAAGAGGAAAAGGAAGATAAAGACAAGCCTAAAGTTGAAGATCTGGATGAGAATGATGAGGATGATGATGATACTTCAAAGAAAGACAAGAAAAAGAAGAAGAAAATCAAGGAAAAGTACAGTGAGGAAGAGGAACTCAACAAAACCAAGCCACTCTGGACTAGGAATTCTGATGACATCACTGCCGAAGAGTATGCTGAATTCTACAAGTCTCTGACCAATGATTGGGAAGACCACTTGGCAGTTAAACACTTTTCTGTGGAGGGACAGTTAGAGTTCAGAGCACTTCTGTTCATCCCAAAGAGGGCGCCATTTGATATGTTTGAGAACAAGAAGAAGAAGAACAACATC**

**Protein (322 aa)**

NTFYSNKEIFLRELISNASDALDKIRYESLTDPSKLDTGKELHIRIIPDKENKTLTIEDTGIGMTKADLVNNLGTIAKSGTKAFMEALQAGADISMIGQFGVGFYSAYLVADRVVVDSKNNDDEQYTWQSSAGGTFTVEPSTSAPLSRGTRVTLYLKEDQLEYLEERKIKDVIKKHSQFIGYPIKLLVEKERDKEVSDDEEEEKKEEEKEDKDKPKVEDLDENDEDDDDTSKKDKKKKKKIKEKYSEEEELNKTKPLWTRNSDDITAEEYAEFYKSLTNDWEDHLAVKHFSVEGQLEFRALLFIPKRAPFDMFENKKKKNNI

**Heat shock protein 17.2 (1690 bp)**

ACCCTGCGTGGAAGTCGTGGTTCTGGATAATTCCCGGGGCCCGGTATAAAACACGGGCTCGCTCTCCCGGACAGTCACAACAAACCGCCTGTACGCTATAAAGTCGTCGCTTACAGCTGAGAAGCATTCATTTTCTAGAGGAACCGTGAACTTGTCGCTTGTTGAGAAGTGCAACAGTAGAAGCGAGA**ATGTTTGCCATCACCCCCCATCGCCGGTACGACGAGTACGACCGTTTATTACGGGTGTTGGACGAGCTGACCAACCCCTGGACCCCGCCTGGCCAGTACTCCGGCGATGGCGCTGTGGCCCCAGTCAGAGGCCGGCTAGGCGACTCTGAGATCCACAACACAGATAAGGAATTCCGCATCCGAATGGATCTGGGCTATTATGCTCCAGAGGAGGTGAAAATCACATCGGATAATGAAAACATTGTTGTCCATGCCAAACATGAAGAGAAACAGGACAACCATGGCTATGTCACACGTGAGATGTGTAGGAAGTACAAGCTACCCAGTGATGTTGACCCTAAGTCGGTGACCTCGACCATGAACTCGCAAGGCATACTCTGCATTAAGGTGCAGAAAAAGATGCTGGAGCAACCAAAGGAGACGGCAATCCCCATTGAGTTTAAGGGT**TAGATTCTGTGCAGTGGTCAGTACAACACACTTGACCAATGCAATCAGCATATCAATACAGGGACAACACACTTGACCAATGTAGTCAACATATAATTATACGGACAACACACTGGACCAATGTACGATTGTTGGTCAATAAAAACGTAGGAAAATCGGAAATATGAACGTGTGATGTAGCAACTAAGTTGGATTAGGCATGACCACAATGTGATGAGTTCGTGGACTTGGATTGACGATCTGACGACAAACCTGAAATGTAGCCAAAAATCGAGCTGAATGATTAAATCCTCAATTAAATATTTAAAATGACCTTAATTTCTTCTCCCAGTGAATTGGTCATTGATTTAAGTTATTGATTTCAGCTGTTTATCTATTCATTATTTAAGATGGTTTTGAAAACATATCTTTTATACATGTAATACTTTATGAATGTGATTAATAGCCTTCGTTTGCTCTGTTTATATTGGTGTTTTAGATACACGCCGTTTCGTTTATGTCCTCTTTTTTCCAAGTTATATATGTTCCCCCTCCAGGCAGGTGTTTGATGGCTGCATATTGTATCACTATCCACGATTAGTTTCCCCCTCTTACCTCAGGAGGCCCCTCCCACTGCTCTGCTCTTCAAACTACTGAACTTAGTTTCTGTTGGGGCTGCATGATACATTTAATGAAAAGTCCATCATAAACTTTCATACTAATCGTATTTAGAGAAGCAAAGGACAGCAAAAAGGTATAAATGAAATGGTAAACAGCTGGGCAGCAACAAAATACAAATTAACTTTATTAATAGGAAGTGGCCGTTAGGATTGGTCAGTCAATGGGACACTAGTAGAAGCAGAATATTCCTTCAGAACATTGACCTGTGATGGATATGTGGTCTGTTTTATTCCCCTTTGGCTTTCATAATTAGGAAAACACCTCACTTGCTTACTTTGTCAGATTTGTTCCTTCCAGCACATGTCAAAAATGTCCCGAAATGCTTATAGCTTCTGTTGCATGATACTTACTAGTCTTGATTTATTTATGTATGTTATTATTACAATTATCCATGC

**Protein (149 aa)**

MFAITPHRRYDEYDRLLRVLDELTNPWTPPGQYSGDGAVAPVRGRLGDSEIHNTDKEFRIRMDLGYYAPEEVKITSDNENIVVHAKHEEKQDNHGYVTREMCRKYKLPSDVDPKSVTSTMNSQGILCIKVQKKMLEQPKETAIPIEFKG

**Heat shock protein 19.8 (1014 bp)**

GAACTCCGCTGTAAGCGATATACGTAGAAGGGAGGAAGTCTATACAAGTGTATTTATTTGCAAAAGAGAAACGTAGTGACTTAGTTTGTGTTAGAAAAGTTTGCCTGAATTGATCACAATTTCAAG**ATGGAACGACTTGTCCCCATTCAGCGAGAAAACTGGAGCTTCTTCGACCGACAGAGGCAAGTCTTCAGCAATCTCTTCAAGGAAGATGATGGCGAGTTTAAGGAATTCGACAAAGAGCTTGAGCGCATTCGAAACGAAATGTTCACACTGAAATCCTCAGACTTCGGCGACGCCAGTTCTACGTATCTCAAGCCCGAACGCCCTATCGTTTCTGACGAGGAGGGAAATAAACGGCTGTCTCTGAGGTTTGACTGCAAAGAATTTAAGCCCGAGGAGATATCAGTGAAAACTGTGGACAACAAGCTGATGGTGCATGCCAAACACACAGAGGAGTCTCCTGGCAGAAAGGTCTACCGTGAATATACACGTCAGTATGTTTTGCCTCAGAAGATTGATCCATTGGCCTTAAAATCAACTTTGGCCTTAGATGGAGTCCTAAGCATCGAAGCACCAGCTCCTGCTACAGTGGAGGCTCCAAGGGAAAGAATTCTTCCTGTAGTGCGTCTG**TAATGTGTCAGTTATCACGATGATTATGTTCAGTGTCTGACAATATTTATATTCAACGTTTTTTTTAAATATTATTGGCTTCAACGTAAAGCAGTTACATATTGAAAAACAAGCTACAAAAAGTCATTGAGCTTTTAACTCATGGGTCCTGGAGACTTCAAAATTCTAGGGCACGAAATCAAACTCACGAGTTCATCTCACGTGAAGTATCGCAGTGGGAAAGGTGTAGGCAAAGGTGAAAATTAGTACCTGGGCGAAGAGAGGGAAAGCCAGAGACAGAAGGAGATAGGGCGAACGATACACACAAAAGAGTAACTTTATTAAATCACGTTTGAAAAATGTATGCAGAACAATTTAGAAGAAAGAATACGACCCACTGCT

**Protein (169 aa)**

MERLVPIQRENWSFFDRQRQVFSNLFKEDDGEFKEFDKELERIRNEMFTLKSSDFGDASSTYLKPERPIVSDEEGNKRLSLRFDCKEFKPEEISVKTVDNKLMVHAKHTEESPGRKVYREYTRQYVLPQKIDPLALKSTLALDGVLSIEAPAPATVEAPRERILPVVRL

**Cu/Zn Superoxide Dismutase (1351 bp)**

GTCAAATTGCACACGGCACAACGCAGCAGAAGTTGCCAAGTCATAGGATTTTCTAGAAAGTCTGCTGCAATTCTCTCTCAAGTTTCTCCAAAATATCACAATTTAAACAGTTAAA**ATGGTGAAAGCGGTTTGTGTTCTTGGTGCAGGAACAGTTTCTACTGTTACAGGAACGATTACTTTCACGCAAGAGAAACTTGGTGATAAAACTGTTGTCTCTGGTGAAGTGAAAGGTCTTACTCCAGGCAATCATGGGTTTCACATTCATGAATTTGGAGACTATACAAACGGCTGTGTTAGTGCTGGAGGACATTTTAACCCTGCAGGCAAGACTCACGGGGGGCCAACTGATACCGAGAGGCATGCTGGCGACCTGGGAAATATAGTTGCCAATGCTGACGGTGTTGCGGTAGTTAATTTGTCAGATGCACAGATTCCCCTTATTGGAGAAAACTCAGTCATTGGTCGTAGTGTTGTGGTCCATGAGAAGGAAGATGACCTTGGCAAGGGTGGCAACGAAGAAAGCTTGAAGACAGGAAATGCTGGCCCTCGCCAGGCGTGTGGTGTGATTGGGGTGACCAAG**TGAACAGACTGAAGTTTAGTGTTTCCGTTCTAGAATATTCTCACAATAGTAACCAGTGAACGACATTAATGTTTAATTATACCTGCTGTTTATGTGAATAATCTGAATCCTGTGGAAGTATTTGATGTAATTTAGAAGTTTTTGTAGAGGTAATCTCATTTTGCATTCCAATTGAGGTGAATTGAAGAAAACTGCTCTCAAATATGTGGTAATAGATTAATGTTCTATAGAATTTTGGAGTATAATTTGTTAATAGACAGTCTCTGGGCATGCCATGAGAAGGGGAGATCTGGAGTATTGTGTCACAAGGAGAAAGTTGGAGGGTAGAAGAAGCATGGGGAGACCAGAAAGAGGATGCTGGACAGTTTGACATCATGGCATAGAGAGACACCGGCATCACAAGAGATAGAAGGCTGTGGACAGACATGATCACCAGCGCCACATGGACCATAAGAAGAACTAATTAATAGTCAATAGGGAAAGGTGAACTAATATAGAATATTATAGATTACTAGTTAAAACCAGCATATTCTCAAAATATTTGTTAATCAACTAAACGCTGTATAGAATTTTGGAGATTTGCTACCTTTAAAAGAAGTTAATGTTGTATAGAAGTTGGTAGTTCAAGTCATTAATACTGAATAACCGTTTCATTGAATTATGCAGCATGATTAATTAATCAATCAACAGCCAGATAGGAACTTTTGTGCTACACAACATTTGTTCTTTAATTGTTTGATCTTGAACATTATAAGTGAATCTTTACAAATG

**Protein (155 aa)**

MVKAVCVLGAGTVSTVTGTITFTQEKLGDKTVVSGEVKGLTPGNHGFHIHEFGDYTNGCVSAGGHFNPAGKTHGGPTDTERHAGDLGNIVANADGVAVVNLSDAQIPLIGENSVIGRSVVVHEKEDDLGKGGNEESLKTGNAGPRQACGVIGVTK

**Mn Superoxide Dismutase (944 bp)**

CCCTGACCTCTAAAGTTGTGCAGACGGCGAGAAGTCGGCAGA**ATGTTGTCAACAACCTCCAACGTTTTGAAAAGTCTGCTTGGAAAGAATGCAGCTCTTGGTGCAGCTACTGCACGACTAAAGCACACATTGCCAGATTTGAAATATGACTTCAATGCTCTGGAACCATATATCTCCGCTGACATCATGAAGCTGCATTACGAAAAGCATCACAATGCATATGTCACAAATCTCAATGTTGCAGAGGAGAAACTGGCAGAAGCTACACAGAAAGGTGACTTGAATGCAATTATCAGTCTCCAACCAGCCCTGAAATTTAATGGTGGAGGACACATCAACCATAGCATCTTCTGGCAGAACTTAAGCCCTAAAGGTGGTGAAGAGCCAGAAGGTGATCTCTTGGCTCAGATCAAAGATGACTTTACTTCATTTGAACGTATGAAGTCTGAACTTGTTGCTGCCTCTGTGGCCATCCAAGGATCAGGATGGGGCTGGCTGGGTTACAGCCCAGTTAATGGCCACTTACGTATTGCTACTTGTGCCAATCAAGACCCTCTTCAAGCGACAACAGGTTTAATACCCTTGTTCGGAATTGATGTTTGGGAACATGCCTACTATCTTCAGTACAAAAATGTGCGACCAGATTACGTGAAAGCAATCTTTCATGTTGCTAACTGGAAAGACGTGTCGGAGCGCTTAGCAAAAGCTCGTTTGGCC**TAATTTGGCAGTATACTAATTAGGTCTAACATGGTTTCGTTATTTTATTGGAAGTTTTAAACATGGGACTAAACGTTAATAAACTGTGAAAATGTCAGTGCTTTGACTTTGTTGAAGGATTCATTCCTTATTGTTGGATTATGTGTCCTGGGTAACTCCTTGTGCACCGAATCTCAAACTCCATAGAGGAAAGCCTTCAACAGTTTAGTTTTTTTTTTTTTTTTTTA

**Protein (225 aa)**

MLSTTSNVLKSLLGKNAALGAATARLKHTLPDLKYDFNALEPYISADIMKLHYEKHHNAYVTNLNVAEEKLAEATQKGDLNAIISLQPALKFNGGGHINHSIFWQNLSPKGGEEPEGDLLAQIKDDFTSFERMKSELVAASVAIQGSGWGWLGYSPVNGHLRIATCANQDPLQATTGLIPLFGIDVWEHAYYLQYKNVRPDYVKAIFHVANWKDVSERLAKARLA

**Bactericidal/permeability-increasing protein (2138 bp)**

GGCGTGTATATGAATAGTGTGTATTCTATATCTGCTATACAAGTTTATTATCAACAATGTTTCTCGATTTCATCTAAGTTTTCATATTTAATACCTCATTGTGTAGCTGCATGGACAGCAGCAATTCCTAATAAATTAATAGCTGCTAATTAACGAGATCTGAACCAAGGCTCGTGTGAGATGGTGAACAACTTTCAGATCACATCCTGAAACATTAATTACAGTTTAAGAG**ATGAGATCACGGACAAGTGACAAGTTGGCAGTATTAATAGTATTTGCCTTTAGTTTGTTAACTTTAACAATGGCAGGAGGGAACCCGGGAGTGCAGATGAGGATAACAGAACGAGGAATTAATTACGTGAACACATTAGCGGAAAATTCTATCAAGGTTGAACTACAAACACTAAAAATAGCTGACCAGTCAGGACAGGAAGGACATTTGTCATGGGAGCTAACAAATATTCAAGTACAAAATGTGGCTGGACCAAGCAGTCAAATAACCTTTAACCCAGAAATGAGTGGTCTGACTTGGTCTCTGTACAATTTCGGCATTAACCTCCGATCCGACTGGCATGTCAAGTTCAAGCAGGGATGGATCAAGATCTCAGACAGCGGCAGCCTGACAGTCGGTCTCAGTGATGTTTCCTTGACTGTCACGGTGGGCTTTGGACAGAAAGATGGCCGCCCATCAATTTCTTCTAAATCCTGCAATGCACACATTGGAAATGTCGATGTTAAATTCCACGGAGGATCTGCCCGTATATTAAATCTGTTCAGGGGAATTGTCGAAGACAGGATTAAAGACTTACTTGAAAGTCGGATGTGTGATGTCTGTAATAAAAAGATTAACCAGGATGTAGAAGAAGCTCTGTCAAAATTAAACGTCTATGTAACCATTGACAACCGTTTTCTCTTGGACTACAGCCTGATAGCGGCCCCCGTTGTCTCAAGCAATTACCTGCAGACATTCAACAAGGGAGAGTTCTACTGGAAAGCTGACATGCAAGAATGCCCGTTTTCACCGAGTGCAATTCCTGAATGGAGCGATGTCAACAGCATGCTCTACATATGGCTAACTGAATACACTGCCAACACGTTTGCTTATCAGGCACATCACCATGGTTACCTCAAATACAACTTGACAAAAGATAACTTGCCGCCGGATAAGGCTGGATACTTAAACACAAGCTGCTCATCTGTCTGTATCGGCACAATCATTAAGCAGATATCTAAAGCTTATCCAAATTCGTGGGTAGAGCTACAATTCAGCTCCACAGCTGTGCCAGCTGTTAAAATCACCAGCCAGAATATAACTGCGGGTGTCCAGGCAAATGTGGATCTAATTGCTCACACACCAGATGGCAGCACTCATTATATTCTCACACTGAACATGAGCTTGACAATGACAGGGCAGGCATCCACTGAAAGAGAGAAACTGATTGCGACAGTTGTAGATCACAGTTTTGATGTCTCAGTGGTGAAATCTGATATTGGAAACATTGATGTTAGAAAAGTGCACATGTTTGTGGAGACCGTGTTGACCTTCTCCATCATCCCAGAACTGAATAAACTTGGCAGAGAAGGCATTGACCTACCAGTGACTCCGAATGTACAGTTTCTGAACACCAAGCTTATCTTGCAGGATGGATTTCTCATGGTGTGCACAGATGCCCAGTACAACAGAAACTTCTACTTTCTACCCGAAATCCAC**TAACACGTGGACCGACCTAACTCCTTCAATTCCTGTGCTCGTCACTCGCTAAGGGCCACCTATTCCTCCAAGCACTGCACCAGAAATATATGAGAGTACATCAAAGATTCATAGCCGCTTCCTGCTACGCAACTTTCCCCCCCTCTCCCCTCAGTTATATTGTATTTCATTTTGTCTTATTTCTCATGTGCAGACAATATGTGATGTGATTTAGCATTTGTTATTATTGTATCTCATTTTGATGATCTTTACCTTATTCTGGTGTACAGGTTACGGACACATTTCGTATTCCCACATTTGAAACAGCTGCAGTTTGGCAGCAGACCACTGTGCATTGCTTTTTTTATGAAATGATATGGGTGCCGGTCATTCAAAAAAGCAATGGCAAACTACTGAACGTAAACTTGCTAAGAGATAACAGCAAACA

**Protein (493 aa)**

MRSRTSDKLAVLIVFAFSLLTLTMAGGNPGVQMRITERGINYVNTLAENSIKVELQTLKIADQSGQEGHLSWELTNIQVQNVAGPSSQITFNPEMSGLTWSLYNFGINLRSDWHVKFKQGWIKISDSGSLTVGLSDVSLTVTVGFGQKDGRPSISSKSCNAHIGNVDVKFHGGSARILNLFRGIVEDRIKDLLESRMCDVCNKKINQDVEEALSKLNVYVTIDNRFLLDYSLIAAPVVSSNYLQTFNKGEFYWKADMQECPFSPSAIPEWSDVNSMLYIWLTEYTANTFAYQAHHHGYLKYNLTKDNLPPDKAGYLNTSCSSVCIGTIIKQISKAYPNSWVELQFSSTAVPAVKITSQNITAGVQANVDLIAHTPDGSTHYILTLNMSLTMTGQASTEREKLIATVVDHSFDVSVVKSDIGNIDVRKVHMFVETVLTFSIIPELNKLGREGIDLPVTPNVQFLNTKLILQDGFLMVCTDAQYNRNFYFLPEIH
